# Inflammation in a ferroptotic environment

**DOI:** 10.3389/fphar.2024.1474285

**Published:** 2024-09-20

**Authors:** Anja Wickert, Anna Schwantes, Dominik C. Fuhrmann, Bernhard Brüne

**Affiliations:** ^1^ Institute of Biochemistry I, Faculty of Medicine, Goethe University Frankfurt, Frankfurt, Germany; ^3^ Frankfurt Cancer Institute, Goethe University Frankfurt, Frankfurt, Germany; ^2^ German Cancer Consortium (DKTK), Partner Site Frankfurt, Frankfurt, Germany; ^4^ Fraunhofer Institute for Translational Medicine and Pharmacology ITMP, Frankfurt, Germany

**Keywords:** HIF, NF-kB, iron, lipid peroxidation, LCN2

## Abstract

Ferroptosis is an iron-dependent form of cell death, which finally culminates in lipid peroxidation and membrane damage. During the past decade, the interest in ferroptosis increased substantially and various regulatory components were discovered. The role of ferroptosis during inflammation and its impact on different immune cell populations is still under debate. Activation of inflammatory pathways such as nuclear factor kappa-light-chain-enhancer of activated B cells (NF-κB) and hypoxia inducible factors (HIFs) are known to alter the ability of cells to undergo ferroptosis and are closely connected to iron metabolism. During inflammation, iron regulatory systems fundamentally change and cells such as macrophages and neutrophils adapt their metabolism towards iron sequestering phenotypes. In this review, we discuss how ferroptosis alters inflammatory pathways and how iron metabolism under inflammatory conditions affects immune cell ferroptosis.

## Introduction

Within the last decades, several forms of cell death were discovered. Besides “classic” forms such as apoptosis and necrosis also pyroptosis and ferroptosis were described. More recently, ferroptosis has gained increasing attention in basic science and clinical settings. First hints of this form of cell demise emerged in the 1950s by H. Eagle who showed that amino acid deprivation increased cell death (Eagle, 1955). The term ferroptosis was coined by Dixon and coworkers in 2012 (Dixon et al., 2012). Ferroptosis depends on iron-mediated lipid peroxidation, which disrupts membrane integrity. In the meantime, various cellular pathways were uncovered to contribute to or prevent ferroptosis. Among these glutathione and iron metabolism are most substantial. Besides cell demise, the contribution of ferroptosis to the pathogenesis of cancer and inflammatory diseases emerged. Interestingly, ferroptosis shares features with inflammation such as an altered iron metabolism and increased oxidative stress. Activation of inflammatory pathways upon infection modulates ferroptosis sensitivity of cells. Inflammation frequently is accompanied by hypoxia, which also affects the oxidative machinery and iron homeostasis and thus, must be considered as an additional link between ferroptosis and inflammation. In this review, we report recent findings on basic ferroptotic mechanisms linking them to inflammatory pathways, iron metabolism, and describe the role of ferroptosis under inflammatory conditions.

## Mechanisms of ferroptosis

Ferroptosis is an iron-dependent form of cell death occurring due to metabolic imbalances, which result in the extensive production of reactive oxygen species and increased lipid peroxide formation, thereby causing membrane damage and cell death. Lipid peroxidation of polyunsaturated fatty acids (PUFAs) is a hallmark of ferroptosis, which was shown to be prevented by respective inhibitors (Bannai et al., 1977; Zilka et al., 2017). During ferroptosis free-radical initiated fatty acid peroxidation as well as 12-lipoxygenase and/or 15-lipoxygenase (ALOX15) facilitated lipid peroxidation may add to membrane destruction. Besides its harmful capabilities, lipid peroxidation mediated by 15-lipoxygenase type B also affects biological processes such as cholesterol metabolism of human macrophages (Benatzy et al., 2024). Moreover, both lipoxygenases use PUFAs such as arachidonic acid, eicosapentaenoic or decosahexaenoic acid to produce specialized lipid mediators with roles in inflammation and wound healing (Zheng et al., 2020; Benatzy et al., 2022).

In a non-enzymatic manner hydroxyl and hydroperoxyl radicals are generated by the Fenton reaction, where free ferrous ions (Fe^2+^) catalyzes the decomposition of hydrogen peroxide (Fenton, 1894). Ferrous iron is highly reactive and is accessible for cellular usage in the labile iron pool (LIP). To overcome iron-mediated cytotoxicity, cells developed a well-orchestrated iron uptake, storage, and release system. In brief, iron is bound to transferrin and taken up into cells via internalization of the transferrin receptor (TfR) (Muckenthaler et al., 2017). Inside endosomes, iron is reduced from ferric to ferrous iron by the metalloreductase STEAP3 and released into the cytosol by the divalent metal transporter 1 (DMT1). Iron storage is mediated by ferritins, which oxidize Fe^2+^ to ferric iron (Fe^3+^) and sequester it. Release of iron from ferritins is achieved by nuclear receptor coactivator 4 (NCOA4) and increases the LIP and thus, sensitized cells towards ferroptosis (Fuhrmann et al., 2020). In turn, cellular export of iron by ferroportin (FPN) protects from ferroptosis (Namgaladze et al., 2022; Geng et al., 2018).

Because of chemical interactions between iron, oxygen, and polyunsaturated lipids, oxidative stress and ROS generation have to be well orchestrated to protect cells from damage. Therefore, defense mechanisms to limit lipid peroxidation are required (Stockwell, 2022). Glutathione peroxidase 4 (GPX4) is a selenoenzyme that directly detoxifies phospholipid hydroperoxides to lipid alcohols in membrane-bound phospholipid peroxides. GPX4 demands glutathione, a cysteine-containing tripeptide, as a cofactor (Ursini et al., 1985). Cells import cystine, the oxidized form of cysteine, through the X_C_^−^ system consisting of solute carrier family 7 member 11 (SLC7A11) mediating cystine/glutamate antiporter activity and solute carrier family 3 member 2, which acts as a chaperone to stabilize the complex in the membrane (Koppula et al., 2021). Further defense mechanisms include ferroptosis suppressor protein 1 (FSP1), which converts phospholipid peroxyl radicals to phospholipid hydroperoxides using ubiquinone and FAD. Thereby FSP1 restricts propagation of lipid peroxidation (Bersuker et al., 2019; Doll et al., 2019). The basic mechanisms of ferroptosis are well reviewed and illustrated, e.g., in ((Berndt et al., 2024), (Stockwell, 2022), (Fuhrmann and Brüne, 2022), (Jiang et al., 2021)).

**FIGURE 1:**
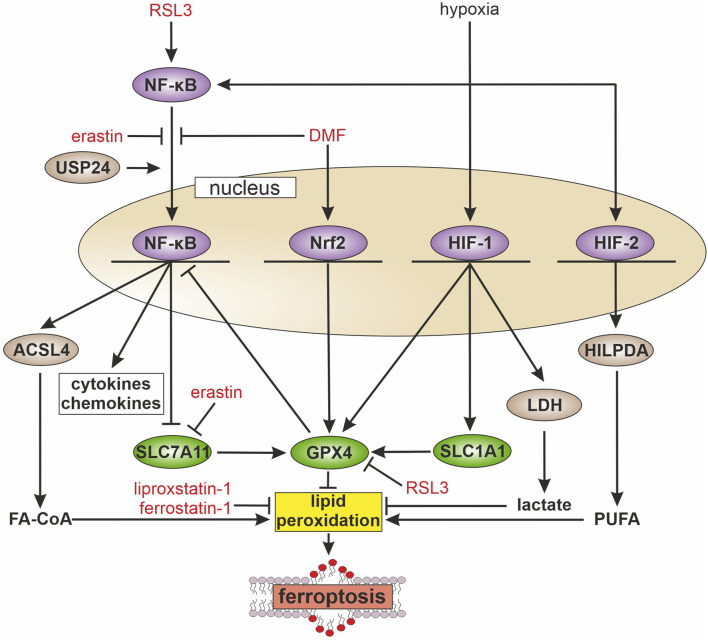
Inflammatory pathways and ferroptosis During inflammation multiple signaling cascades are activated, e.g., the transcription factors nuclear factor kappa-light-chain-enhancer of activated B cells (NF-κB), nuclear factor erythroid 2-related factor 2 (Nrf2), and hypoxia-inducible factor (HIF). NF-κB is activated by ras-selective lethal small molecule 3 (RSL3) and the ubiquitin-specific protease 24 (USP24), while erastin and dimethyl fumarate (DMF) block its activation. Activated NF-κB increases the transcription of long-chain-fatty-acid-CoA ligase 4 (ACSL4), which facilitates fatty acyl CoA (FA-CoA) synthesis and ferroptosis. Further, NF-kB blocks the expression of solute carrier family 7 member 11 (SLC7A11), with consequences for glutathione synthesis and glutathione peroxidase 4 (GPX4) activity, which abolishes lipid peroxidation. In contrast, Nrf2 increases GPX4 expression and thus, attenuates ferroptosis. HIF-1 enhances solute carrier family 1 member 1 (SLC1A1) expression, which indirectly supports GPX4 activity. Further, HIF-1 protects from ferroptosis by elevating glycolysis, expression of lactate dehydrogenase (LDH), and facilitating lactate production. HIF-2 acts pro-ferroptotic by increasing hypoxia-inducible lipid droplet-associated protein (HILPDA) and polyunsaturated fatty acid (PUFA) release, which in turn increases lipid peroxidation.

During the last decade, various inducers and inhibitors of ferroptosis were developed (Figure 1). Ferroptosis inducers are predominantly blocking defense mechanisms, such as the GPX4 inhibitor Ras-selective lethal small molecule 3 (RSL3) or erastin, which lowers cystine supply by inhibiting the X_C_^−^ system. Most inhibitors of ferroptosis antagonize lipid peroxidation, for example, radical-trapping antioxidants, such as liproxstatin-1 and ferrostatin-1 or they reduce the LIP, acting as iron chelators like deferoxamine (Zilka et al., 2017).

## Ferroptosis and inflammatory signaling

Under inflammatory conditions multiple signaling cascades affect transcriptional patterns that facilitate cytokine and chemokine expression, modulate resolution of inflammation and regulate ferroptosis-associated pathways (reviewed in (Chen Y. et al., 2023)). While this review focusses on nuclear factor kappa-light-chain-enhancer of activated B cells (NF-κB), hypoxia inducible factors (HIFs), and iron, other inflammatory mediators have been connected to ferroptosis as well. For example, tyrosine-protein kinase JAK (JAK)/signal transducer and activator of transcription (STAT) signaling, regulates the X_C_^−^ system. Activation of JAK/STAT signaling by interferon γ decreased solute carrier family 3 member 2 and SLC7A11 expression, thereby facilitating ferroptosis of adrenocortical and hepatocellular carcinoma cells (Yu et al., 2022; Kong et al., 2021). In addition, interferon γ signals via interferon regulatory factors, which caused expression of SLC7A11 and GPX4, thereby protecting from ferroptosis (Liu et al., 2023). Also, aryl hydrocarbon receptor needs consideration, which regulates SLC7A11 (Kou et al., 2024). Pharmacological and genetic inhibition of aryl hydrocarbon receptor decreased SLC7A11 and sensitized human normal bronchial epithelial cells for erastin-induced ferroptosis. In the following chapters of this review we describe the complex interplay between NF-κB, HIF, iron, and ferroptosis.

## Implications of NF-κB for ferroptosis

**FIGURE 2:**
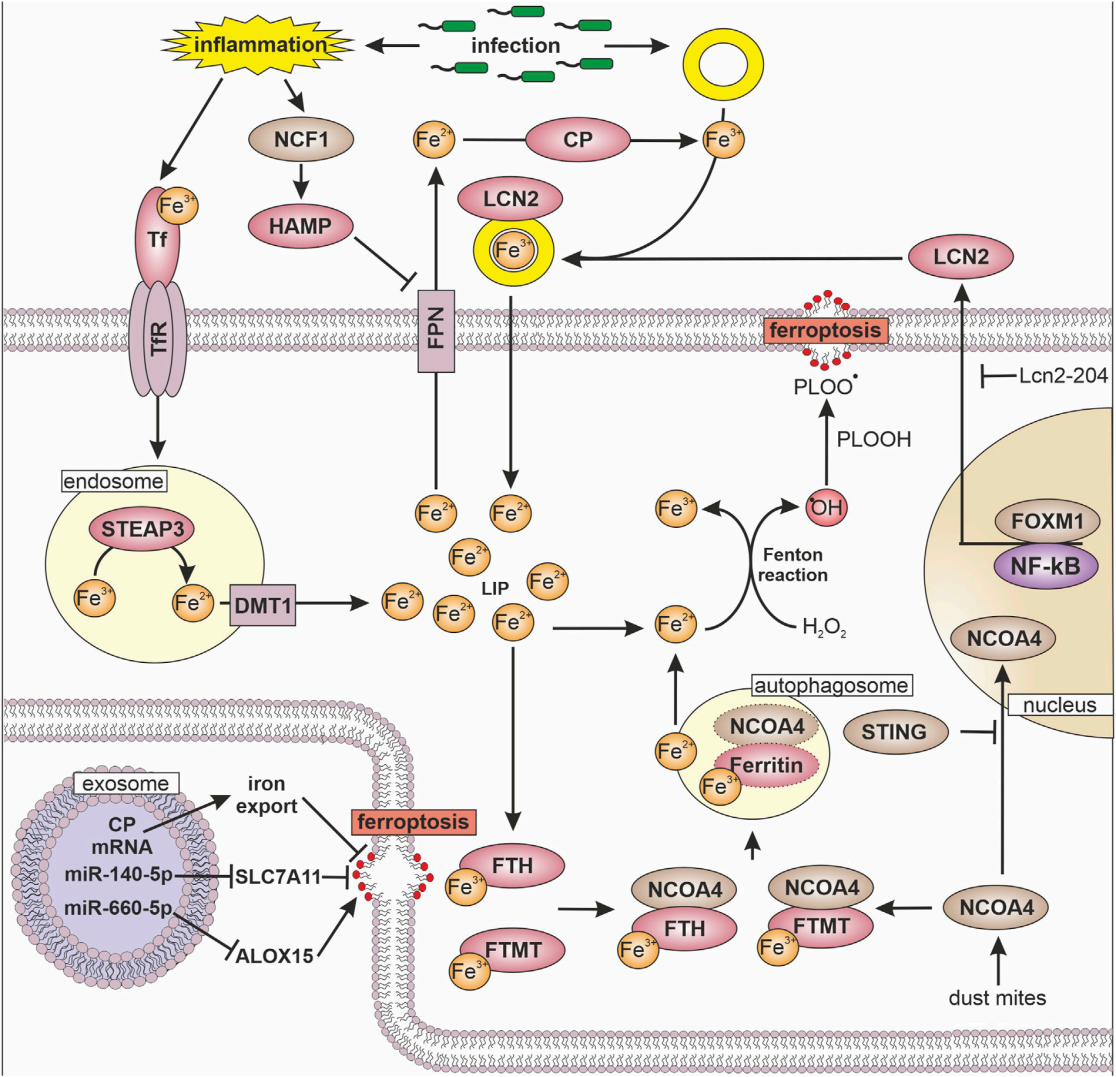
Iron, inflammation, and ferroptosis Transferrin (TF) bound iron (Fe) is internalized into the cell via transferrin receptor (TfR). In endosomes metalloreductase STEAP3 reduces iron (Fe^3+^ → Fe^2+^) followed by divalent metal transporter 1 (DMT1) mediated release into the cytosol, where it forms the labile iron pool (LIP). To avoid hydrogen peroxide (H_2_O_2_) mediated formation of hydroxyl radical (^•^OH) and conversion of phospholipid hydroperoxide (PLOOH) to phospholipid hydroperoxyl radicals (PLOO^•^), ferritin heavy chain (FTH) and mitochondrial ferritin (FTMT) oxidize and store iron. To release iron from ferritin, nuclear receptor coactivator 4 (NCOA4) marks FTH and FTMT for autophagosome-mediated degradation, which increases the LIP. The transfer of NCOA4 to the nucleus is inhibited by the stimulator of interferon genes (STING). Another way to sequester iron is via lipoclain 2 (LCN2), which is expressed dependent on forkhead box protein M1 (FOXM1) and nuclear factor kappa-light-chain-enhancer of activated B cells (NF-κB), while long non coding RNA Lcn2-204 inhibits LCN2 expression. LCN2 binds siderophore (yellow ring) bound iron and thereby reduces iron availability for pathogens. Export of iron from cells is facilitated by ferroportin (FPN) in close cooperation with ceruloplasmin (CP), which enables iron binding to TF and siderophores. FPN is regulated by hepcidin (HAMP), which is increased by neutrophil cytosolic factor 1 (NCF1) upon inflammatory stimuli. The ability to undergo ferroptosis can be modulated by exosomes, which transfer micro RNAs (miR) or mRNAs to target cells. Following their uptake miRs regulate the expression of solute carrier family 7 member 11 (SLC7A11) and 15-lipoxignase (ALOX15).

Ferroptosis plays a role in several physiological and pathophysiological processes such as tumor suppression or aging. Recent studies revealed that ferroptosis is also involved in inflammation. A hallmark regulator of inflammation is the transcription factor NF-κB. The transcription factor is activated by various stimuli such as pathogen antigens, cytokines or genotoxic stress (Perkins, 2007). The active homo- or heterodimeric NF-κB complex consists of p65, p50, and p52. These components are constantly expressed but kept inactive by binding to NF-κB inhibitor alpha (IκBα). To activate the transcriptional activity, IκBα is degraded upon phosphorylation by IκB kinase. Afterwards, NF-κB translocates to the nucleus and induces the expression of pro-inflammatory genes including cytokines and chemokines (Bonizzi and Karin, 2004). Recently, several studies explored how ferroptosis affects NF-κB signaling and whether NF-κB regulates ferroptosis susceptibility (Figure 1). Yao and coworkers show that the loss of leukemia inhibitory factor receptor in liver cancer activated NF-κB, which upregulates the iron-sequestering protein lipocalin-2 (LCN2), thereby decreasing ferroptosis sensitivity (Figure 2) (Yao et al., 2021). Vice versa, septic shock in mice is ameliorated by pretreatment with the ferroptosis inducer erastin. Mechanistically, erastin treatment of bone marrow-derived macrophages reduces phosphorylation of IκB kinase β and consequently phosphorylation and degradation of IκBα. This prevents nuclear translocation of NF-κB in lipopolysaccharide (LPS)-stimulated murine macrophages and decreases the production of inflammatory mediators such as nitric oxide, tumor necrosis factor-α, and interleukin-1β (Oh et al., 2019). In some analogy, the group of Li observed that GPX4 activation inhibits TNF-mediated activation of the NF-κB pathway in HEK293T cells (Li et al., 2018). In experiments with chronic cerebral hypoperfusion in mice, treatment with the multiple sclerosis drug dimethyl fumarate (DMF) decreases pro-inflammatory cytokines via the NF-κB pathway and mitigated oxidative stress in the hippocampus (Yan et al., 2021). In parallel, DMF treatment reduces the iron content, likely by elevating ferritin heavy chain (FTH) expression and increasing glutathione levels by enhancing cystine import via the system X_C_^−^ in hippocampus, which potentially protects cells from ferroptosis and reduces hippocampal neuron injury (Figure 2). Inhibition of the NF-κB pathway by DMF is facilitated by activation of the antioxidative transcription factor nuclear factor erythroid 2-related factor 2 (Nrf2), which decreases ferroptosis sensitivity. Besides Nrf2, also Nrf1 protects from ferroptosis by upregulating GPX4. Activation of Nrf1 demands a cytosolic peptide:N-glycanase 1, which causes its deglycosylation (Forcina et al., 2022). In addition, Nrf1 sustains proteasomal activity and thereby protects from ferroptosis likely by preventing GPX4 hyper-ubiquitination (Kotschi et al., 2022). Further, interactions between ferroptosis and NF-κB were discovered in glioblastoma cells, where RSL3 activates NF-κB. Moreover, inhibition of NF-κB mitigates RSL3-induced ferroptosis (Li et al., 2021). To explore the impact of NF-κB in executing ferroptosis, GPX4 was silenced but an additional activation of the NF-κB pathway was necessary to effectively induce ferroptosis in glioblastoma cells. These studies indicate an important role of NF-κB in executing ferroptosis. In line, a recent study shows that upregulation of the ubiquitin-specific protease 24 (USP24) in myocardial cells activates the NF-κB pathway in diabetic cardiomyopathy (Wu et al., 2024). This activation decreases RNA expression of the ferroptosis suppressors SLC7A11 and FTH and increases expression of the ferroptosis promoter long-chain-fatty-acid-CoA ligase 4 (ACSL4), pointing to the involvement of NF-κB and hence ferroptosis in the pathogenesis of diabetic cardiomyopathy.

## Interactions between hypoxia inducible factors, inflammation, and ferroptosis

Inflammation is often accompanied by hypoxia, which arises when oxygen demands exceeds its supply (Taylor and Colgan, 2017). To ensure sufficient energy production, cells adapt to hypoxia by activating transcriptional regulators, known as HIFs. HIFs comprise two subunits, HIF-α and HIF-β. While HIF-β is constantly expressed, the HIF-α subunits are hydroxylated by prolyl hydroxylases and degraded under normoxic conditions. Under hypoxia, HIF-α is stabilized, translocates to the nucleus, dimerizes with the β-subunit, and facilitates target gene expression to modulate, e.g., cellular metabolism, angiogenesis, and erythropoiesis (Semenza et al., 1991; Semenza, 2012). Under inflammatory conditions, NF-κB activates hypoxic signaling even under normoxia by increasing the expression of HIF-1α and -1β (van Uden et al., 2008; van Uden et al., 2011). Further, accumulation of metabolites like succinate upon LPS stimulation add to the stabilization of HIF (Fuhrmann et al., 2018). Conversely, HIF-1 facilitated activation of NF-κB in neutrophils (Walmsley et al., 2005). This effect was confirmed in transgenic mice with a gain of HIF-1 function, which increased activation of the NF-κB pathway (Scortegagna et al., 2008). As inflammation and HIF-signaling are closely connected, the question remains whether HIF affects cellular sensitivity towards ferroptosis. Indeed, studies by Yang et al. observe ferroptosis-protecting effects upon HIF-1 activation (Yang et al., 2023). Mechanistically, HIF-1α enhances glycolysis and lactate dehydrogenase (LDH) expression (Figure 1). Thereby, lactate increases and protects the cells from ferroptosis in a pH-dependent manner. Moreover, HIF-1α increases the expression of the glutamate-transporter solute carrier family 1 member 1 (SLC1A1) that promotes cystine uptake and therefore ameliorates ferroptosis resistance. In line with these findings, the ferroptosis-protective effect of HIF-1α was confirmed in non-small cell lung cancer, where HIF-1α was upregulated as seen in many types of cancer. Silencing HIF-1α increases ROS and Fe^2+^ levels and decreases glutathione and GPX4. Furthermore, the absence of HIF-1α enhances cell death, which was partially prevented by the ferroptosis inhibitor ferrotstatin-1 (Zheng et al., 2023). In contrast, HIF activation was also observed to sensitize cells towards ferroptosis. Inhibition of HIF-α degradation increases ferroptosis sensitivity. Distinct inhibition of HIF-1α or HIF-2α reveals that HIF-2α induces genes, which are involved in lipid metabolism, contribute to excessive lipid peroxidation and therefore increase ferroptosis sensitivity (Su et al., 2023). The ferroptosis-sensitizing effect of HIF-2α was also observed in clear cell carcinomas, where HIF-2α selectively enriches polyunsaturated lipids via upregulation of the hypoxia-inducible lipid droplet-associated protein (HILPDA) (Zou et al., 2019). Summarizing these studies, HIF-1α is described to be ferroptosis-suppressive, while HIF-2α sensitizes cells towards ferroptosis. One of the major differences of HIF-2α compared to HIF-1α are increased protein levels upon long-term hypoxia (Holmquist-Mengelbier et al., 2006; Fuhrmann et al., 2015). Thus, under acute hypoxia, HIF-1α plays a more important role than HIF-2α, thereby contributing to decreased ferroptosis. Under chronic hypoxia, HIF-2α levels increase and provoke higher ferroptotic susceptibility.

Recapitulating, under acute inflammation and/or hypoxia NF-κB and HIF-1α activate each other. Predominantly, HIF-1α increases anti-oxidative pathways to enhance ferroptosis resistance. However, NF-κB facilitates ferroptosis-sensitizing effects. Depending on the cell type and stimulus, HIF-1α and NF-κB show either cooperative or contrary effects on ferroptosis susceptibility. This interdependent relationship might regulate the balance between cell survival and cell death in inflammation. However, under chronic hypoxia stabilization of HIF-2α shifts cellular metabolism to a pro-ferroptotic state, which potentially results in excessive tissue damage.

## Iron, inflammation, and ferroptosis

Besides its crucial function for cellular integrity, iron represents a link between hypoxia, inflammation, and obviously ferroptosis. Both, hypoxia and inflammation induce an iron scavenging phenotype in macrophages upon their activation by extracellular stimuli, e.g., by inducing or resolving inflammation. Under inflammatory conditions, macrophages reduce blood iron levels to limit the availability of this factor for pathogens (Marques et al., 2022). Inflammatory macrophages sequester iron by increasing iron storage via ferritin and decrease FPN-mediated iron export, while alternatively activated macrophages release iron by elevating the amount of FPN and LCN2 (Jung et al., 2015). In patients with rheumatoid arthritis M2 like macrophages show higher lipid peroxidation and ferroptosis compared to M1 macrophages, which were likely protected by their increased ability to store iron (Liu et al., 2024). Blocking ferroptosis by liproxstatin-1 increases anti-inflammatory M2 macrophage populations and alleviates arthritis progression in mice.

To remove iron during infection, macrophages increase their TfR to facilitate iron uptake. Besides TfR, macrophages and neutrophils express LCN2, which binds to bacteria-derived siderophores and therefore limits iron availability for pathogens by scavenging siderophore-bound iron (Figure 2) (Jung et al., 2017). Under septic conditions, neutrophil-derived LCN2 induces ferroptosis in cardiomyocytes by increasing the labile iron pool and lipid peroxidation (Huang et al., 2022). In part, the long non-coding RNA Lcn2-204 is responsible for ferroptosis in cardiomyocytes in sepsis. Silencing Lcn2-204 reduces LCN2 expression as well as iron overload and provokes a cardioprotective and anti-ferroptotic effect (Huang et al., 2024). Based on a machine learning approach, LCN2 is considered as biomarker for sepsis-induced acute respiratory distress syndrome (Zhan et al., 2024). In ulcerative colitis, LCN2 expression correlates to elevated lipid peroxidation and decreased GPX4 expression (Deng et al., 2023). Silencing LCN2 in this model suppresses ferroptosis. In contrast to inflammatory conditions, LCN2 protects renal and colorectal tumor cells from ferroptosis by decreasing intracellular iron levels and increasing GPX4 expression as well as causing Nrf2 activation (Chaudhary et al., 2021; Meier et al., 2021). Furthermore, silencing LCN2 sensitizes T-cell acute lymphoblastic leukemia cells to RSL3-mediated ferroptosis (Tian et al., 2023). Expression of LCN2 in endometrial cancer is facilitated by forkhead box protein M1 (FOXM1) (Jiang et al., 2023). Therefore, silencing FOXM1 decreases LCN2 and increases ferroptosis, which is eliminated by LCN2 overexpression. In breast cancer cells, a knockout of LCN2 increases the sensitivity towards cisplatin and stimulates ferroptotic cell death (Valashedi et al., 2022). These studies underscore an ambivalent role of LCN2, which protects cancer cells from ferroptosis, while it acts pro-ferroptotic under inflammatory conditions.

In the blood, most iron is bound to transferrin and is endocytosed upon binding to the transferrin receptor. After internalization, iron is reduced to Fe^2+^ by the metalloreductase STEAP3 and released from endosomes via DMT1. Under inflammatory conditions, iron uptake increases in conjunction with an attenuated release, due to a lower FPN surface expression as a result of hepcidin (HAMP) evoked degradation of the iron exporter (Nemeth and Ganz, 2021). Substantial amounts of HAMP are produced in the liver but also monocytes and macrophages release small quantities to regulate their own FPN expression. These mechanisms favor intracellular iron accumulation and uncontrolled iron-mediated production of reactive oxygen species and potentially, lipid peroxidation. To opt out, macrophages developed efficient iron storage systems. Ferritins increase under inflammatory and hypoxic conditions and can be considered as acute phase proteins during inflammation (Marques et al., 2022). Ferritins oxidize free iron and store it in spheres built of ferritin light chain, FTH, or mitochondrial ferritin (FTMT). FTMT shares high sequence homology with FTH but contains a mitochondrial target sequence, which is removed in macrophages by thrombin-mediated cleavage under hypoxic or oxidative stress, including LPS treatment. Thereby cells increase their ability to scavenge iron and protect themselves from lipid peroxidation (Fuhrmann et al., 2023), a mechanism operating in macrophages to circumvent ferroptosis. Experiments in human macrophages show that the sensitivity towards RSL3-mediated ferroptosis increases after the knockdown of FTH and/or FTMT (Fuhrmann et al., 2020). In this setting, hypoxia decreases NCOA4 expression, which reduces lysosomal degradation of ferritin, a process termed ferritinophagy. Thereby, the release of iron into the labile iron pool was diminished and consequently, protection against ferroptosis increased. A higher rate of ferritinophagy, going in line with enhanced lipid peroxidation and ferroptosis, was evident in a house dust mites-induced asthma model (Zeng et al., 2022). Mice exposed to house dust mites show increased signs of inflammation, which decrease in the presence of deferoxamine and ferrostatin-1. Mechanistically, increased NCOA4 expression goes in line with enhanced free iron, lipid peroxidation, and ferroptosis. Conclusively, ferroptosis of airway cells induces inflammation in mice, which implies that ferroptosis can either result in or be a result of inflammation as seen in another model of acute LPS-induced lung inflammation which induces ferroptosis. In this setting, meteorin-like/meteorin-β protected from ferroptosis by inhibiting p53 and increasing SLC7A11 expression (Chen Z. et al., 2023). Ferritinophagy is also connected to sepsis, where NCOA4-mediated ferroptosis contributes to disease severity by increasing inflammation. Mechanistically, stimulator of interferon genes (STING) blocks nuclear translocation of NCOA4, which facilitates ferritinophagy, iron release, and lipid peroxidation (Wu et al., 2022). Sepsis is often accompanied by cardiac dysfunction caused by the inflammatory response. Ferrostatin-1 suppresses lipid peroxidation and ferroptosis upon LPS-induced cardiac inflammation in rats and improves sepsis-induced cardiac dysfunction (Xiao et al., 2021). Underlining the pivotal role of STING under inflammatory conditions, STING promotes hepatic iron accumulation in mice suffering from autoimmune hepatitis (Zhao et al., 2024). Besides increasing ferritinophagy, STING regulates iron release as shown by a liver-specific knockdown of STING. In this setting STING ameliorates iron accumulation and oxidative stress induced by increased expression of neutrophil cytosolic factor 1 (NCF1), which promotes HAMP expression in liver cells (Zhang et al., 2024). HAMP indirectly, by degrading FPN, provokes an iron overload in Kupffer cells, with consequences for ferroptosis, inflammation, and metabolic dysfunction-associated steatohepatitis (MASH). Another example for the interaction of macrophages with their environment was found in a tumor context, where macrophages invade the tumor and are polarized to tumor-associated macrophages. Apparently, tumor-associated macrophages protect tumor cells from RSL3-mediated ferroptosis (Schwantes et al., 2024) because these macrophages release extracellular vesicles containing ceruloplasmin mRNA. Once taken up by tumor cells, the mRNA is translated into ceruloplasmin protein, which supports iron export with lower rated of lipid peroxidation and ferroptosis. Moreover, tumor-associated macrophages protect tumor cells by an IL-13/IL-4-mediated increase of miR-660-5p, which was packed into exosomes and transferred to tumor cells (Luo et al., 2023). MiR-660-5p blocks ALOX15 expression and reduces lipid peroxidation in tumor cells, which attenuates ferroptosis. In contrast to tumor-associated macrophages, adipose tissue macrophages release exosomes containing miR-140-5p, which reduces SLC7A11 expression (Zhao et al., 2022). A lower amount of SLC7A11 enhances lipid peroxidation and mitochondrial dysfunction by decreasing cystine import and consequently, glutathione synthesis.

Taken together, iron metabolism links inflammation and ferroptosis. Inflammatory conditions alter cellular iron homeostasis and thereby their ferroptotic susceptibility. The other way round, ferroptosis supports inflammation by recruiting and activating immune cells.

## Conclusion

During the last decade, ferroptosis gained attention in basic and applied sciences, being connected with various disease conditions. Ferroptosis as of now emerges as a therapeutic target but whether it has to be induced or inhibited strongly depends on the context. To generalize, induction of ferroptosis appears favorable in cancer, while its inhibition attenuates severe inflammation. Under inflammatory conditions, ferroptotic cells evoke immune responses including cytokine production and immune cell attraction. Unfortunately, which immune cells are sensitive to ferroptosis and to which extent is still under debate. Further, the role of ferroptosis inducers, e.g., erastin or RSL3 in inflammation appears to be ambivalent, which may point to off-target effects. While RSL3 promotes NF-κB signaling, erastin rather causes it inhibition. In the future, analyzing the interplay between lipid peroxidation, ferroptosis, and inflammation will increase our understanding of basic signaling principles. While ferroptosis can easily be studied in cell culture, only a few studies have demonstrated an essential role in humans or mice. Therefore, the impact of ferroptosis towards the cross-communication of immune cells with parenchymal cells needs careful evaluation for its *in vivo* relevance. Still, it is hoped that acquired knowledge may open avenues that help to direct ferroptosis under various disease conditions in either promoting or attenuating this from of cell demise.
